# Engaging frontline workers in policy development to support the implementation of measurement-based care: Lessons learned from a field-based workgroup

**DOI:** 10.3389/frhs.2022.929438

**Published:** 2022-08-08

**Authors:** Rajinder Sonia Singh, Sara J. Landes, Sandra G. Resnick, Traci H. Abraham, Kelly P. Maieritsch, Stacey J. Pollack, JoAnn E. Kirchner

**Affiliations:** ^1^United States Department of Veterans Affairs, Center for Mental Healthcare and Outcomes Research, Central Arkansas Veterans Healthcare System, Veterans Health Administration, Little Rock, AR, United States; ^2^Department of Psychiatry, University of Arkansas for Medical Sciences, Little Rock, AR, United States; ^3^Behavioral Health QUERI, Central Arkansas Veterans Healthcare System, North Little Rock, AR, United States; ^4^Northeast Program Evaluation Center, Office of Mental Health and Suicide Prevention, U.S. Department of Veterans Affairs, New Haven, CT, United States; ^5^Department of Psychiatry, School of Medicine, Yale University, New Haven, CT, United States; ^6^Executive Division, United States Department of Veterans Affairs, National Center for Posttraumatic Stress Disorder, Hartford, VT, United States; ^7^Office of Mental Health and Suicide Prevention VA Central Office, Washington, DC, United States

**Keywords:** policy, measurement-based care, policy implementation, posttraumatic stress disorder, policy development

## Abstract

Measurement-Based Care (MBC) is the use of patient-reported outcome measures repeatedly over the course of treatment to track progress and empower both providers and patients to collaboratively set goals and plan treatment. The Measurement-Based Care in Mental Health Initiative within the Department of Veterans Affairs' Office of Mental Health and Suicide Prevention partnered with the Post Traumatic Stress Disorder (PTSD) Mentoring Program to create an interdisciplinary field-based workgroup. The workgroup included psychologists, clinical social workers, and mental health counselors from PTSD Clinical Teams. The task of the workgroup was to create guidelines for best practice in delivery of MBC in PTSD Clinical Teams given anticipated policy requiring MBC to be used in PTSD Clinical Teams. Framed in the Strategic Action Field Framework for Policy Implementation Research, the current paper evaluates this hybrid top-down and bottom-up process of policy development. Major barriers included difficulty with the workgroup as an authentic bottom-up process, inability to reach the entire field (e.g., focus groups not widely attended by providers), and limited diversity in the workgroup. Facilitators included using consensus to make decisions, support provided to workgroup members by national operations partners, and collaboration and mutual respect among workgroup members. Workgroup members noted an equal, respectful partnership between operations partners and the workgroup; they reported feeling empowered and believed the viewpoints of the field were included in the guidelines. Further, due to the COVID-19 pandemic, the workgroup included more guidelines specific to telehealth into the guidelines. This hybrid model provides a process through which frontline workers can inform policy development and implementation.

## Introduction

Measurement-based care (MBC) is the use of patient-reported outcome measures repeated over the course of treatment to track progress and empower both providers and patients to collaboratively set goals and plan treatment (1, 2). MBC refers to making decisions about clinical care on the basis of these data and is becoming widely used in mental health care (3). As a leader of providing quality healthcare in the United States, the Veterans Health Administration (VHA) has worked to implement MBC through providing trainings, resources, and placing effort into successful implementation into VHA's mental health programs, including residential and outpatient programs (4). Moreover, VHA created an initiative specific to the dissemination and implementation of MBC referred to as the MBC in Mental Health Initiative (which will be here on referred to as the MBC Initiative). The initiative provides training and technical assistance related to MBC and uses a “collect, share, act” paradigm, in which providers are encouraged to collect data via self-report measures, share and discuss the results of these measures with the patient, and act through shared decision making and treatment planning with the veteran patient (4, 5).

In recent years, VHA has begun a multiyear effort to increase MBC use in mental health through a series of phased roll outs (6). Phase 1 of implementation was described as a “learning by doing” process that informed efforts to expand use of MBC in VHA and focused on Champion Sites that volunteered to implement MBC (5). Phase 1 focused on these 59 volunteer sites including 185 participating clinics primarily representing substance use disorder clinics, outpatient PTSD clinics, general mental health clinics, primary care mental health integration, residential rehabilitation treatment programs, and a small number of other programs such as medical psychology, inpatient, and vocational programs (5). The evaluation of the Champion Sites in Phase 1 highlighted several barriers and facilitators to implementing MBC in VHA. Barriers included limited time and technology; concerns about use of measures; and lack of staff, clinician, and leadership buy-in (5). Facilitators included site champion and staff engagement, availability of technology, and use of MBC to inform care (5). The majority of clinics that performed best in implementing MBC had leadership support on the local, regional, and national level, as well as mandates for their clinics to use MBC (4).

The MBC Initiative entered Phase 2 following the 2018 Joint Commission mandate for MBC in programs accredited under Behavioral Health standards. The MBC Initiative continued to provide training and technical assistance to programs including those that had not participated in Phase 1. Mental Health Residential Rehabilitation Treatment Program programs and outpatient specialty substance use disorder treatment programs were both included in the Joint Commission mandate; therefore, leaders in the MBC Initiative assisted program leaders with creating policy for the implementation of MBC in these program areas. During Phase 2, providers from the Home-Based Primary Care program began organizing for implementation of MBC in Home-Based Primary Care without a specific mandate that required this group to do so. Providers in Home-Based Primary Care created a workgroup consisting of frontline workers (i.e., psychologists,), surveyed the field about their use of MBC, and drafted guidelines for implementation of MBC based on input from the field. This grass-roots organizing and implementation of MBC by Home-Based Primary Care is an example of an authentic bottom-up process in which workers in the healthcare field lead implementation efforts. Typically in VHA, changes to healthcare processes are driven from the top-down, so this unique bottom-up process led to MBC Initiative leaders decision to use this workgroup model in Phase 3 and to support the creation of program-specific guidelines across VHA.

The MBC Initiative entered Phase 3 in January 2020. A goal of this phase is to provide national guidelines for use of MBC across the mental health continuum. In this phase, the MBC Initiative is creating program-specific field-based workgroups to ensure that input from the field (i.e., frontline workers and program managers) informs national implementation efforts. These groups create guidelines that provide practical tools for the field to assist with local implementation of MBC, recommendations for MBC best practices, and these guidelines may influence policy development in the VHA. In any healthcare system, policy is the priorities set by the system including the formal laws, procedures, regulations, administrative actions, incentives, or voluntary practices that influence the healthcare system (7). The push toward MBC at the national level in VHA indicates it as a priority of the healthcare system.

### Measurement-based care in PTSD clinical teams and workgroup creation

**Figure 1:**
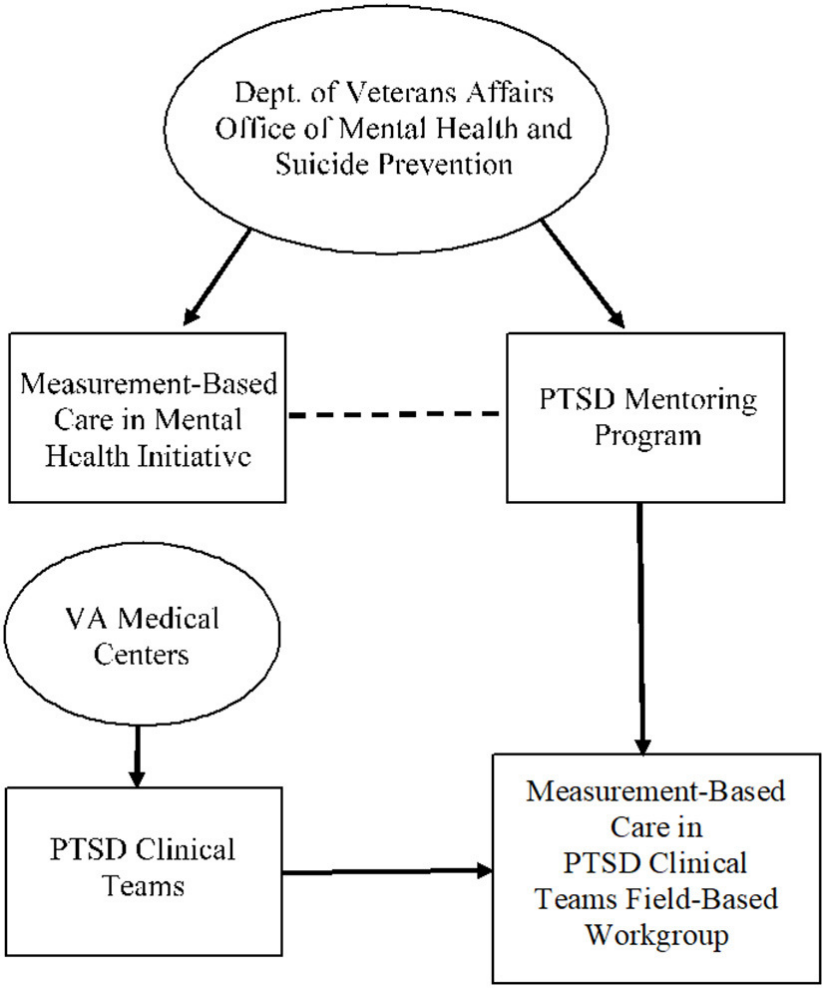
Structure of VA operations partners and field-based workgroup members.

PTSD Clinical Teams are specialty clinics throughout VHA designed for the mental health treatment of veterans who are experiencing PTSD or other trauma sequalae. Upon entering Phase 3, the MBC Initiative chose to begin with PTSD Clinical Teams in their process of creating implementation guidelines through engaging frontline workers to create a field-based workgroup. There are several reasons the MBC Initiative began this workgroup process with PTSD Clinical Teams. As part of the MBC Initiative, the Office of Mental Health and Suicide Prevention wished to create policy requiring PTSD Clinical Teams to implement MBC. Moreover, many PTSD Clinical Teams have a strong emphasis on evidence-based treatments for PTSD that require the collection of measures throughout treatment. Further, the PTSD Mentoring Program, a national consultation and training program specific to PTSD throughout VHA, provides training on MBC, encourages the use of MBC, and has familiarity with providers who use MBC. Figure 1 provides an overview of structure of the MBC in PTSD Clinical Teams Field-Based Workgroup.

The PTSD Clinical Teams Field-Based Workgroup was tasked with developing recommendations to the Office of Mental Health and Suicide Prevention in the form of guidelines for the use of MBC in PTSD Clinical Teams. These guidelines would be used as an MBC implementation tool and for PTSD Clinical Teams to use as a resource for how to best adhere to forthcoming policies toward use of MBC. The workgroup was encouraged to use all available data to form recommendations (e.g., literature, feedback from the field). Operations partners encouraged the workgroup to consider the variability of services provided across PTSD Clinical Teams, different provider disciplines, and the needs of providers balancing competing demands. The MBC in PTSD Clinical Teams Field-Based Workgroup was also encouraged to discuss use of specific measures, frequency of collection, and best practices for all three areas of MBC (i.e., collect, share, and act).

### MBC in PTSD clinical teams field-based workgroup process

To form the MBC in PTSD Clinical Teams Field-Based Workgroup, first the MBC Initiative partnered with the PTSD Mentoring Program and personnel in the Office of Mental Health and Suicide Prevention with extensive knowledge of PTSD Clinical Teams because of their familiarity, knowledge, and connection to the field. The PTSD Mentoring Program invited potential workgroup members who they believed would represent various disciplines and include providers who had varying viewpoints related to MBC. The PTSD Mentoring Program also nominated someone to chair the workgroup. The potential members and chair accepted the invitations and the MBC in PTSD Clinical Teams Field-Based Workgroup (hereafter referred to as workgroup) was formed.

The initial kickoff meeting for the workgroup was in March 2020. Initially, the workgroup was to meet for a 2-day intensive meeting to draft the guidelines to be set in April 2020. However, the COVID-19 pandemic began spreading in the United States quickly after the kickoff meeting. This resulted in the workgroup pausing and the delay of guideline development. In April 2020, the workgroup met to discuss the stability of the field and decided to move forward with creating guidance.

The workgroup was provided several existing documents through the MBC Initiative to assist in the process of writing the guidelines. In their process of soliciting feedback from the field, the workgroup collaboratively created a survey instrument that asked questions related to MBC (e.g., How often do you administer measures in your setting? What are the typical measures you use in your setting?). They publicized their survey through PTSD Mentoring Program calls and listservs. The workgroup members used survey results to guide the development of an interview guide for focus groups. The workgroup conducted focus groups at varying times during a span of 2 weeks consisting of drop-in groups that any provider could join. These focus groups were advertised via the PTSD Mentoring Program. The workgroup integrated survey results, focus groups, and previous MBC documents. Next, workgroup members collaborated to draft guidance and assigned portions of the guidelines to different workgroup members, with the chair largely outlining and overseeing the process. Finally, the workgroup sent a full draft of the guidelines to operations partners for their edits and approval.

Operations partners including the PTSD Mentoring Program, MBC Initiative, and additional representation from the Office of Mental Health and Suicide Prevention, reviewed the suggested guidelines and provided feedback and edits. Operations partners also solicited feedback from additional stakeholders in the Office of Mental Health and Suicide Prevention. The guidelines were revised based on feedback from operations partners and additional stakeholders before the final guidelines were approved and disseminated to the field. As a result of this hybrid bottom-up and top-down process of policy development, in November of 2020, a memorandum requiring that PTSD Clinical Teams increase implementation of MBC was disseminated to the field. The guideline from the MBC in PTSD Clinical Teams Field-Based Workgroup was referenced as an independent document within this memorandum.

### Strategic action field framework for policy implementation

Given that this workgroup process was a hybrid bottom-up and top-down policy implementation process, measurement design and evaluation were guided by the Strategic Action Field Framework for Policy Implementation Research (8). In this framework, policy implementation systems are conceptualized as multilevel strategic action fields that form around an intervention. A strategic action field is a social order in which actors (individual or collective) interact with each other and have a common understanding about the purposes of the strategic action field, the relationships in the strategic action field (e.g., who has power), and rules governing legitimate action in the strategic action field (9). The boundaries of strategic action fields are fluid and emergent, and develop through participants engaging in shared work, determining their roles and relationships, and creating an understanding of their goals and rules of what is acceptable or unacceptable (9).

There are three overarching components of the framework, including (1) the program intervention (i.e., the thing being implemented by the policy), (2) the scale of analysis (multiple levels where strategic action can occur), and (3) drivers of change and stability. Each component includes various elements that can result in variation in implementation. See Table 1 directly from Moulton and Sandfort (8) for the components, elements, and examples of variation that can result from these elements.

**Table 1:** Strategic action field framework for policy implementation research components.

**Component**	**Elements**	**Examples of variation**
***Program intervention***		
	Processes of change	Degree of complexity as indicated by routinization, number of steps, or predictability; targeted change (e.g., people changing vs. people processing)
	Methods of coordination	Degree of reliance on technical expertise; variation in the sequencing of tasks (e.g., sequential, pooled, interdependent); tools in use with varying coerciveness, directness, automaticity, and visibility
	Change in system operations	Alterations in processes used by agency (e.g., efficiency, accessibility), as well as degree of integration of intervention into everyday practices (normalization)
	Change in target group behavior or conditions	Alterations in target group experiences, as well as degree of change in their behavior or conditions
***Scale of analysis***		
	Policy field (assembly)	Types of structures in use, historical relationships, newness of the field
	Organization (operationalization)	Degree of intervention alignment with other program processes and technologies
	Frontlines (enactment)	Degree of worker discretion; degree of engagement with the target population
***Drivers of change and stability***		
	Sources of authority	Degree of (perceived) influence from political authority, economic authority, norms, beliefs, and values
	Social skills	Degree of use of tactics such as interpreting, framing, brokering, and bridging
	Exogenous shocks	Degree of stability or instability; changes in funding, legislation, or field actors

*From Moulton and Sandfort (8), p. 148*.

The multilevel strategic action fields in the framework include (1) policy field, (2) organizational, and (3) frontlines (8). Each level operates in its own strategic action field in which actors possess varying power, play particular roles, and share a broad interpretive frame that guides action within the field at the level (8). The policy field level is the topmost level in which resources and understanding flow top-down. The policy field level also includes the historical context of the system and actors who create policy. The next level is the organizational level. It is at this level, which includes institutions that link between the policy field and the frontline levels, where operational decisions are executed. The final level is the frontlines, which includes the actors who interact directly with the target group and carry out the policy at the ground level. At each level, the drivers of change and stability component (i.e., sources of authority, social skills, and exogenous shocks) creates the rules and dynamic boundaries around action in relation to the program intervention (8). As such, the Strategic Action Field framework provides an integration of top-down and bottom-up approaches.

### Evaluation of the MBC in PTSD clinical teams field-based workgroup

The Strategic Action Field framework provided the conceptualization for the evaluation and served as a guide for interview questions for workgroup members who worked at different levels of the healthcare system (e.g., PTSD Clinical Team providers, operations partners who work in national VHA offices). The Strategic Action Field Framework allowed for the assessment of different factors associated with guideline development, as well as workgroup members and operations partners' perception of the workgroup process. The purpose of this paper is to describe the process of the workgroup, strengths and difficulties the workgroup encountered, and use these findings to provide recommendations for how to improve and use this process to engage frontline workers (i.e., direct care professionals) in policy development in complex organizations.

## Methods

As stated above, evaluation of the workgroup and data collection were guided by the Strategic Action Field Framework for Policy Implementation Research (8). Data collected for this evaluation included qualitative interviews and live workgroup meeting observations. The evaluation team conducted baseline and follow up interviews with six participants (three workgroup members and three operations partners) for a total of 12 qualitative interviews and 14 observations of workgroup meetings. Recordings of qualitative interviews were transcribed, and detailed notes were taken during workgroup meetings. All qualitative data were initially analyzed using template analysis to capture content and then organized into summary templates.

### Participants and procedures

Potential interview participants included three operations partners associated with VHA Office of Mental Health and Suicide Prevention with knowledge of MBC and/or PTSD services and nine workgroup members who were mental health providers in PTSD Clinical Teams. For recruitment, an email was sent requesting volunteers to participate in semi-structured interviews by telephone. Of the nine workgroup members, three expressed interest in being interviewed. All three operations partners expressed interest in participating. All project procedures were approved by the first author's institutional review board and the project received a non-research determination.

#### Qualitative interviews

A total of 12 semi-structured interviewers were conducted. Six semi-structured interviews lasting 30–60 min were conducted prior to beginning workgroup activities, and six semi-structured interviews lasting 30–60 min were conducted after the conclusion of the workgroup.

Qualitative interviews were conducted by telephone or Microsoft Teams. Interviews were audio recorded and transcribed verbatim. A semi-structured interview guide informed by the multi-level components from the Strategic Action Field Framework was used to solicit the experiences of the operation partners and workgroup members, as well as their thoughts on the workgroup process, what was helpful or unhelpful about the workgroup, and recommendations for future workgroups and implementation of MBC.

#### Workgroup observations

The workgroup met virtually for 14 meetings. During this time, the workgroup collected information from the field (e.g., survey sent to PTSD Clinical Teams, focus groups conducted with PTSD Clinical Team clinicians), collaboratively drafted guidelines, and finalized guidance to share with operations partners. Focused observations guided by the Strategic Action Field Framework were conducted at each virtual workgroup meeting. To record these observations, the evaluator took detailed notes, which formed the basis for qualitative analysis.

### Data analysis

Qualitative interview and workgroup observation notes were analyzed using template analysis, a data reduction technique developed by VHA health services researchers (10). This method involves development of a template, which is subsequently used to summarize and organize interview content into domains and categories (11). As this method allows the analyst to define domains pertinent to project goals a priori, it is well-suited to partnered projects, which often require rapid turn-around to inform policy and practice. It is more efficient than long-standing qualitative methods (12).

Interview transcripts and workgroup observation notes were analyzed with a focus on the workgroup process. First, a summary template was created containing broad, deductive domains informed by components from the Strategic Action Field Framework. Thus, template domains corresponded with questions on the interview guide, focusing analysis on project goals to make analyses more efficient. The primary analyst read through each interview transcript and observation note, populating each template domain with interview content. The primary analyst developed inductive categories within each domain to describe content.

All interviews were templated by the primary analyst. To ensure the completeness and accuracy of templates, 33% of the interview transcripts were independently templated by a secondary analyst and any discrepancies between template content were reconciled through consensus. Templated observation notes were audited by a second analyst for accuracy and completeness.

## Results

Findings from 12 key informant interviews and 14 workgroup observations are organized by the three Strategic Action Field for Policy Implementation Research components: Program Intervention (Field-Based Workgroup); Scale of Analysis; and Drivers of Change and Stability. Illustrative quotes are provided below to illustrate select categories within these components. A summary of all categories identified through template analysis is presented in Supplementary Table 1.

### Program intervention—Field-based workgroup process

Overall, the workgroup was able to effectively work together through methods of coordination (e.g., equality on the team, shifting tasks between members). Barriers included uncertainty related to the state of the field and use of MBC, concerns about how to engage providers who had difficulty with MBC or had limited time in providing feedback about their thoughts related to MBC. Facilitators included using consensus to make decisions, support provided to workgroup members by national operations partners, and collaboration and mutual respect among workgroup members.

In one meeting, a workgroup member expressed concern about adequately reaching providers who have limited time:

“*One of the things that we talked about [is engaging] people where it's harder to pick up measurement-based care. Those are the people who are going to be less likely to call into a meeting or answer a poll. I think those people are just staying above water. If there's a way we can reach out to them and talk to them, that's going to give us helpful information. I would like to target those groups who are typically underrepresented and underserved.”* (Meeting 05/06/20)

Another workgroup member noted how workgroup members were able to shift tasks and rely on each other:

“*I do think there was a nice kind of shift between work group members, some people picked up at one point and then others would jump in kind of later. So, some people seemed like they really had the time to crank something out in this really short span and once they did I felt like I could jump in on the next part and really equal out the contributions thought the timing may have not been the same*.” (P02 Follow-Up Interview)

### Scale of analysis

Regarding scale of analysis (i.e., different levels involved in the workgroup process and changes/adjustments made at those levels), analysis identified difficulties with communication (e.g., workgroup unaware of or missing historical context of MBC, difficulties with reaching diverse participants for workgroup) and the difficulty of achieving an authentic, bottom-up implementation process as barriers. Facilitators included workgroup members expressing novelty and freedom of bottom-up process, engaging existing channels for workgroup (i.e., PTSD Mentoring Program), as well as effective partnership between operations partners and workgroup members.

One operations partner noted how they wished the process could have been even more bottom-up by attempting to cast a wider net and engaging a broader diversity of frontline workers to participate:

“*I wanted it - it would've been even more bottom-up. I would have put out a call to the field and asked for volunteers. [We] would have specifically looked for people that nobody had heard of or worked with to try to get more diversity and perhaps to even have it be a little less circumscribed*.” (P06 Baseline Interview).

Despite this barrier, workgroup members noted the novelty and freedom of the workgroup process:

“*This is sort of revolutionary to have guidance like this come from the bottom up, so to have team leaders and program coordinators be the ones writing these guidelines as opposed to coming down from VA central office […] The fact that we get to formulate them and discuss them and when they figure out what would work best and what's reasonable and what's doable is really wonderful*.” (P01 Baseline Interview)

### Drivers of change and stability

Finally, the drivers of change and stability (i.e., forces that are assisting or blocking the workgroup process) component examines which constructs may be influencing or creating change. Findings indicated barriers to the workgroup process in this component included operations partners ultimately having the power in guideline approval, difficulties due to the COVID-19 pandemic and the workgroup not being able to meet in person, and a lack of protected time and resources of workgroup members. Facilitators included the collaborative, respectful working relationship between the workgroup members and operations partners, group cohesion of the workgroup, and the workgroup continuing to consider the best needs of the field related to the COVID-19 pandemic.

## Discussion

To implement MBC throughout VHA, there has been a multiyear effort to increase MBC use in mental health through a series of phased roll outs. Phase 1, the champion site phase, focused on learning from sites effectively implementing MBC. Phase 2 occurred due to the Joint Commission mandate for MBC in specific programs. The MBC Initiative entered Phase 3 with the goal of institutionalizing MBC across VHA and creating program-specific guidelines for mental health programs within VHA. The MBC Initiative is moving forward with this via field-based workgroups made up of providers who actively work on the frontlines with veterans. Given the anticipated policy change for PTSD Clinical Teams to require MBC, Phase 3 began with the MBC in PTSD Clinical Teams Field-Based Workgroup. Workgroup members were selected and invited via their existing relationship with the PTSD Mentoring Program. Workgroup members worked collaboratively and drafted guidelines by splitting up sections and reviewing as a team. Upon completion of guidelines, the document was reviewed by operations partners in the Office of Mental Health and Suicide Prevention, revisions were made, and subsequently a memorandum was sent to the field that stated MBC is required in PTSD Clinical Teams and made available the guidelines as an independent document to demonstrate best practice for MBC in PTSD Clinical Teams.

Difficulties during the workgroup process included a lack of knowledge regarding the current state of the field in respect to MBC, MBC resources, and historical context related to MBC, as well as difficulties recruiting diverse stakeholders within the workgroup. Further, although the workgroup drafted the guidelines, ultimately, it was operation partners who reviewed and approved them, resulting in the perception that the process was more top-down than bottom-up. Finally, the COVID-19 pandemic changed the logistics of the workgroup and delayed the workgroup process.

Despite these barriers, the workgroup was able to effectively work together through methods of coordination (e.g., equality on the team, shifting tasks between members). Workgroup members noted the novelty and freedom of bottom-up process of guideline development as well as spoke to an effective partnership between operations partners and the workgroup. Workgroup members noted feeling empowered by the workgroup process and a belief that the viewpoints of the field were being included in the guidelines. Further, due to the COVID-19 pandemic, the workgroup included more guidelines specific to telehealth providers into the guidelines.

### Limitations

Although there are multiple strengths of the current work, it is not without limitations. One limitation includes the sample size. We sampled all of the available operations partners and 33% (three out of nine) of the workgroup members; however, typically a sample size of 12 is recommended to reach data saturation in qualitative data analysis (13). We believe workgroup observation data provided additional information toward saturation. The potential of sampling bias of those who selected to be interviewed is also a limitation. Further, given the limited sample size these data are not generalizable and replicative evaluations of future workgroups should be conducted.

### Opportunities for future research

To address these limitations, it would be beneficial to conduct additional evaluations of similar workgroups and assess similarities and differences. Further, it would be beneficial to evaluate frontliner workers who did not volunteer for or participate in workgroup activities (e.g., focus groups, survey) or did not know about the workgroup at all in order to access gaps in reach of the workgroup. Evaluating the perceptions of those who were not reached would highlight the needs of people who may not be as easily accessible or less likely to use the guidelines from the workgroup. Finally, quantitative measurement of use of MBC in PTSD Clinical Teams would be beneficial to assess the differences prior to and after the implementation of the field-based workgroup guidelines.

### Recommendations for future or similar stakeholder work groups

Based on qualitative interviews and observational data, the following recommendations are made for future workgroups or similar workgroups:

#### Organize relevant resources and encourage review by workgroup

One benefit of the MBC in PTSD Clinical Teams Field-Based Workgroup process was that there were available MBC resources including best practices, guidelines, empirical articles, and a charter related to the workgroup. It would be beneficial to organize and provide resources related to best practices and encourage workgroup members to review these documents prior to and throughout the workgroup when necessary.

#### Have a flexible and available operations partner

One of the operations partners was consistently flexible and responsive regarding timeline and well-being of workgroup members. Further, this operations partner responded well to the context of the workgroup meetings. For example, the operations partner was typically a silent participant unless specifically addressed. This allowed workgroup members to ask questions amongst themselves and solve their own problems in the development of the guidelines. However, it was also helpful to have the operations partner on the call to answer questions related to logistics, design of the survey, and any higher-level operations information of which workgroup members were unaware. Having operations partners available or an individual who has higher level contextual information related to the healthcare organization and is willing to respond flexibly would be helpful for similar workgroups.

#### Recruit diverse stakeholders

Workgroup members and operations partners suggested a wider recruitment effort for the workgroup in order to include providers of varying disciplines who may be underrepresented or have varying opinions about MBC. Including patients as key stakeholders in guideline development was also suggested. It would be beneficial to allow several weeks and potentially even months to try to recruit workgroup members of various disciplines, backgrounds, and points of view to increase diversity in the workgroup.

#### Frame communication from the workgroup to the field in language reflecting the overall field's values of providing effective care

Workgroup members consistently used empowering language when addressing the overall field to inform frontline workers that they were actively involved in shaping the field in a meaningful way through their input.

#### Create tools to survey the field

This workgroup used consensus to decide on questions used to survey the field (i.e., the survey and focus groups). Typically, workgroup leadership drafted these questions, but survey and focus group questions were actively discussed during meetings and reviewed by workgroup members before finalization.

#### Partner with other programs or offices to advertise surveys or focus groups to the field

The workgroup partnered with the PTSD Mentoring program to send out their survey and advertise their focus groups. Although they noted this does not reach all providers, they encouraged members of the PTSD Mentoring program to send this information to their teams. Their survey results reached all of the VHA regional networks in the nation. It may also be beneficial to engage other methods of recruitment to ensure people in more diverse areas are being reached (e.g., partnering with healthcare organization leadership at different levels, distribution via listservs specific to the area of interest).

#### Use feedback from the field to check guideline development

Workgroup members reviewed data from the survey and focus group and incorporated these data into their guidelines. It is important to check data and ensure the workgroup is considering all data from the field and creating guidelines based on best practices as well as integrating thoughts and considerations from the field.

#### Divide labor and create deadlines

This group collaboratively worked together with people taking separate sections of the guidelines. Further, there were specific deadlines for when to review and return feedback. Edits to the guidelines were also discussed during meetings. If necessary, leadership would encourage workgroup members to complete a task in real time during the meeting.

#### Use regular (virtual) meetings

Workgroup members responded well to weekly meetings to create guidelines. Some voiced preferring this method along with uncertainty that creation of the guidelines could have been completed within 2 days of in person meeting. Workgroup members noted appreciating the time between meetings to thoughtfully consider and work on guidelines, as well as the flexibility allowed by virtual meetings. Some group members also noted a desire for more informal social interaction which could be integrated into virtual meetings. One group member noted that it is possible the in person process would have been more efficient because it would have allowed for a more timely completion of the guidelines within the original 2 day time frame and edits thereafter. In addition to increase in efficiency, the in person process would allowed for workgroup members to connect on a more personal level with one another as they worked on the guidelines.

#### Continue mindfulness of the COVID-19 pandemic

Workgroup members and operations partners were aware of and responsive to the needs of the field during the beginning of the COVID-19 pandemic. Further, as a result of the pandemic, the workgroup shifted to strongly consider needs for MBC while providing telehealth. As the effects of the COVID-19 pandemic likely continue to unfold for years to come, it is important the workgroups be aware of shifting needs in healthcare as a result of the pandemic and respond accordingly.

### Implications for policy development and implementation

This process of policymakers engaging frontline workers in a field-based workgroup was truly novel. The result of this workgroup included specific guidelines for best practice related to the use of MBC in PTSD Clinical Teams that were drafted by the workgroup and reviewed by operations partners. Some of the unique factors that contributed to this was workgroup members being invested in MBC and understanding of best practices of MBC. Further, workgroup members worked together collaboratively and cohesively. The workgroup members also felt very supported by the operations partners. Through this process, workgroup members and the field at large, felt empowered and heard through the creation of a document related to how to best implement evidence-based care designed by those who are the end users of the policy.

One particular reason this process may be useful is the involvement of “street-level bureaucrats” (14). Street-level bureaucrats are frontline workers who directly interact with the recipients of policy (e.g., patients) and have the discretion to make decisions about their duties. In this case, they make the decision as to whether they implement MBC in their individual level work or not. Street-level bureaucrats have a large impact on the public and the extent to which policy is accurately implemented (14). Policy implementation is improved when street-level bureaucrats are able to work with greater levels of personal discretion in their jobs and when they report increased willingness to implement a new policy (15). Past research has highlighted that when street level bureaucrats report increased willingness, their use of personal discretion to implement policy also increases (16). Street level bureaucrats' willingness to implement policy is increased by understanding policy goals, professional knowledge related to policy, and ability to critically evaluate the policy (17).

These findings suggest that guidelines created by frontline workers or street-level bureaucrats may increase the willingness, understanding, and knowledge of other frontline workers as they carry out policy in their settings. Thus, it is possible that using a field-based workgroup to create guidelines would lead to an improvement in policy implementation at the frontlines. Organizations could benefit from a process such as this to create documents that are designed by and approved of the frontline workers who are expected to adhere them. Future research should examine the impact of involving frontline workers in policy creation on implementation and uptake of policy.

## Data availability statement

The raw data supporting the conclusions of this article will be made available by the authors upon request to the corresponding author.

## Author contributions

RS contributed to project design, data collection, interpretation of results, and writing of manuscript. SL and SR contributed to project design and writing of manuscript. TA contributed to project design, interpretation of results, and writing of manuscript. KM and SP contributed by providing project input and input on the organization and editing of the manuscript. JK contributed to project design and methods and writing and editing of manuscript. All authors contributed to the article and approved the submitted version.

## Funding

This work was supported by the Department of Veterans Affairs Office of Mental Health and Suicide Prevention. RS was supported by the Department of Veterans Affairs Office of Academic Affiliations Advanced Fellowship Program in Mental Illness Research and Treatment and the Department of Veterans Affairs South Central Mental Illness Research, Education, and Clinical Center (MIRECC). SL was supported by a Quality Enhancement Research Initiative (QUERI) grant (PII 19-462), the South Central MIRECC, and the Translational Research Institute (TRI), UL1 TR003107, through the National Center for Advancing Translational Sciences of the National Institutes of Health (NIH).

## Conflict of interest

The authors declare that the research was conducted in the absence of any commercial or financial relationships that could be construed as a potential conflict of interest.

## Publisher's note

All claims expressed in this article are solely those of the authors and do not necessarily represent those of their affiliated organizations, or those of the publisher, the editors and the reviewers. Any product that may be evaluated in this article, or claim that may be made by its manufacturer, is not guaranteed or endorsed by the publisher.
