# Supplementary material for: Engaging frontline workers in policy development to support the implementation of measurement-based care: Lessons learned from a field-based workgroup
Source: Front Health Serv. 2022 Aug 8;2:929438. doi: 10.3389/frhs.2022.929438 (PMC10012816; doi:10.3389/frhs.2022.929438)
Supplement: Supplementary file 1 [file Table_1.DOCX]

# Supplementary Table 1.

*Examples of Barriers and Facilitators to Implementation of Measurement-Based Care organized by the Strategic Action Field for Policy Implementation Framework*

| ***Framework Component***  and **Study Elements**  (with definition) | | Barriers | Facilitators |
| --- | --- | --- | --- |
| ***Program Intervention*** |  | |  |
| **Processes of Change:**  Degree of complexity as indicated by routinization, number of steps, or predictability; targeted change  (e.g., people changing vs. people processing) | - Uncertainty about what MBC actually looks like in the field   *“We want to learn what is actually happening in the field to make sure we are not all the way in left field, but we also want to suggest best practice guidelines*” (M 05/20) | | - Workgroup came to a consensus of a minimum of how frequently to collect measures based on field feedback   *“Should we say the PTSD Checklist (PCL-5) and the Patient Health Questionnaire (PHQ) as a minimum? It does look like [based on the survey] more people use the PHQ other than others. I was thinking about the feedback from the focus group about people wanting to choose their own additional measures. I think that gives us more flexibility.”* (M 07/22) |
| **Methods of Coordination:**  Degree of reliance on technical expertise; variation in the sequencing of tasks (e.g., sequential, pooled, interdependent); tools in use with varying coerciveness,  directness, automaticity, and  visibility | | - Timeline pushed back   The timeline was pushed back twice due to unforeseeable circumstances. | - Workgroup members felt supported by MBC Initiative and PTSD Mentoring Program   “I think the support that we received from the national MBC initiative as well as the PTSD mentoring program.” (P 02F)   - Interdisciplinary team/everyone viewed as equals   “I love that we were an interdisciplinary team, I also feel like there was no pressure for anyone to be an expert.” (P 01F)   - Workgroup members shifted tasks/picking up slack between members   “I do think there was a nice kind of shift between work group members, some people picked up at one point and then others would jump in kind of later so some people seemed like they really had the time to crank something out in this really short span and once they did I felt like I could jump in on the next part and really equal out the contributions thought the timing may have not been the same.” (P 02F)   - Front line providers have leeway to decide guidelines   *“I get the impression that we’re going to have a lot of leeway with these guidelines. One other exciting component of it is […] we’ll be collecting feedback from our providers.”* (P 01B)   - Virtual, regular meetings were helpful   *“I think having regular or more frequent meetings would be helping. Having a chance to come back together and doing some more of that.”* (M 05/06)   - Different workgroup members took lead on different task - Scheduling telephone lines for focus groups - Drafting emails - Included language in sending out poll that group would solicit further information - Coupling sending out poll with mentoring call - Coordination of focus groups - Tracked attendance of Focus group - Took notes and sent out themes - Setting deadlines for workgroup   *“We need three deadlines. First, we’ll assign parts, get the first draft, comments, and deadline to revise your section based on comments, then pulling it all together.”* (M 06/24)   - Virtual meeting was helpful   *“I think it worked virtually better than I thought I would so that was great.”* (M 08/05)  *“I don’t know how we would have done this all in two days. It would’ve been a lot.”* (M 08/05)   - Divided writing of the guidelines into sections - Everyone responsible for their own sections - Everyone reviewed and provided feedback on sections |
| **Change in system operations:**  Alterations in processes used by agency (e.g., efficiency, accessibility), as well as degree of integration of intervention into everyday  practices (normalization) | |  |  |
| **Change in target group behavior or conditions:**  Alterations in target group (providers) experiences, as well as degree of change in their behavior or conditions | | - Workgroup members expressed concern about engaging people who find it hard to use MBC   *“One of the things that we talked about for people where it’s harder to pick up measurement-based care. Those are the people who are going to be less likely to call into a meeting or answering a poll. I think those people are just staying above water. If there’s a way we can reach out to them and talk to them that’s going to give us helpful information. I would like to target those groups who are typically underrepresented and underserved.”* (M 05/06)   - Some focus groups were not attended by any providers   *“I know [focus groups] were not well attended - that people did not typically join those calls.”* (P 04F) | - Workgroup reviewed survey results which matched their assessment indicating receiving accurate feedback from field   *“Overall, it sounds like the feedback we are thinking about in here is matching up with what we are seeing in the poll. I think it would be helpful for us to think about in the focus group – what do we need to know from the field? […]. I think this is the opportunity to get less structured with people because the poll was very structured and we also want to make sure we are answering any questions we need to have answered for the guidelines.”* (M 07/01) |
| ***Scale of Analysis*** | |  |  |
| **Policy field (assembly):**  Types of structures in use, historical relationships, newness of the field | | - Workgroup members were unaware of/missing contextual history of MBC   *“Some of the questions you are having, we had some of these questions about Phase I. We had these discussions. You are going to find pros and cons and you have to make decisions about pros and cons. From the experience we had, you’re going to have a lot of information and one way to distill it is to sort through how you are going to distill it.”* (M 05/06)   - Operations partners expressed concern that information from frontlines may not always filter up to VA Central Office   *“At a facility where there’s a very, very strong hierarchical philosophy or negative repercussions for going out of your chain of command, it is possible that those mechanisms [to provide feedback] while we think they are available, but they actually aren’t because there is too much fear about contacting [VACO].”* (P 06B) | - Bottom-up guideline development is a new process   *“Your document is going to be the bottom-up thing. Here’s why we think – all the things you’ve been talking about. […] You’ve already come from a different perspective than I would. I think there’s things that you want central office to know. I know all of you have been on the receiving end of documents that are telling you what to do and think about that, manual or paper. What elements have been helpful to you to you. […] One of the best things I’ve seen about how you’ve been operating is you’re thinking strategically, thinking about what is important to your peers, don’t lose that by trying to model what’s been done before.”*  (M 06/17)   - Workgroup members expressed trust in the PTSD Mentoring Program   “*I think that the PTSD mentoring program in particular is a reason why PTSD mentees, mentors often feel empowered right, often feel we have that support behind us and really there was this expression of how much value the mentoring program felt like this would bring to the field.*” (P 02F)   - Existing relationships of PTSD Mentoring Program with MBC Initiative were helpful   “*My experience it was initially being approached by [operations partner] to update where the initiative was going and talking about wanting to focus on these field-based groups and asking about if PTSD would be a great place to start. And within our mentoring program we have a structure for connecting people in leadership positions who are already change agents, so it made a lot of sense to partner and pull together the workgroup from our participants in our program.*” (P 03F)   - Channels already existed to have information go up and down from PTSD mentoring program   “*The general messaging that consistently comes from the PTSD mentoring program in my opinion is vital.*” (P 02F)   - Guideline development was an open-ended process based on workgroup’s own expertise   “*It was very open-ended. There was no pressure to kind of have it mirror some other guidelines for some other program. I felt like it was purely based on our own understanding of what we researched and read and the feedback that we received from the front line. We weren’t persuaded in anyway and that was refreshing.*” (P 01F)   - Operations partners provided resources, but not pressure   “*There are a ridiculous number of resources related to MBC. We gave references so the workgroup wasn’t starting from scratch by any means. I sent some articles, I sent a presentation, I said use whatever you can. We gave them the resources but they are the ones who combed through and put together the guidance from those resources.*” (P 03F)   - Operations partners specifically reached out to workgroup members to be available/show support   “*So, a lot of people in our program, I would reach out and ask “How is it going? How are you doing it?” and they would often respond, “I can’t talk about it right now, but knowing you’re there, is helpful.” So I think that’s the sort of the same approach we took to the workgroup.*” (P 03F) |
| **Organization**  **(operationalization):**  Degree of intervention alignment with other program processes and technologies | | - Workgroup members were unaware of resources for telehealth and existing MBC resources - Constant changing technology in VHA hindered guideline development   “*We had a number of different technologies but it’s gotten more complicated because each one works in different ways and each one has something different for the patient and provider and it wasn’t until just about after the guidance had been done that another application came out – Mental Health Checkup – that seems to be a solution for all the things. However, its new tech and it has issues and we are trying to figure that out.*” (P 03F) | - Workgroup members believed they were creating guidelines for effective care   “*[Let’s] frame it in the context that PTSD teams are all really rebranding themselves right now. People are looking more at those levels of care and us being super specialized care and what’s so important about being super specialized care, well, you’re doing a type of care that’s really effective and you’re being involved in making sure the care is effective.*” (M 06/17)   - Facility viewed provider’s involvement in workgroup as important and an honor   “*At the local level they recognize like this is a really important project that’s going to make changes nationwide and it’s an honor to be a part of it, so I was really happy with that.*” *(P 01F)* |
| **Frontlines (enactment):**  Degree of worker discretion; degree of engagement with the target population | | - Concern workgroup did not receive input from all frontline providers by only advertising on PTSD mentorship call   “*One downside is a lot of the field isn’t on that call, so it’s mostly mentors and mentees and not necessarily all the frontline staff so we wouldn’t get those folks.*” (M 06/17)   - Low rate of medical provider responses to poll   “*There were not a lot of med providers which may because in general due to a lack of med providers in PCTs.*” (M 06/17)   - Could be even more bottom-up   “*I wanted it - it would’ve been even more bottom-up. I would have put out a call to the field and asked for volunteers. [We] would have specifically looked for people that nobody had heard of or worked with to try to get more diversity, and to have it, and perhaps to even have it be a little less circumscribed.*”  (P 06B) | - Workgroup expanded times focus groups were offered to increase access for frontline providers   “*If each of us had a specific time, give everyone a [telephone] line a use for those slots if they wanted to and it’s a drop in call. Anyone can join the call. We’ll offer several times.*” (M 05/27)   - Used PTSD Mentorship call to advertise poll   “*[Partner] is very open to using mentoring group to get the word out. […] We are thinking mentors would forward it to their focus, send to mentoring group and mentoring group would send to their staff.*” (M 06/10)   - Used PTSD Mentorship call to advertise poll   **“***We have to be sensitive to being too authoritarian, you know that these are guidelines and these are recommendations that think we want to be mindful that some people who are already struggling if we make the lines you are, you must do this every single time then we won’t bring them on board. So thinking about our goal of routine measurement we may be less prescriptive about how routine that is.*” (P 02B)   - Workgroup members felt empowered in development of guidelines   “*This is sort of revolutionary to have guidance like this come from the bottom up so to have, to have team leaders and program coordinators be the ones writing these guidelines as opposed to coming down from VA central office […] The fact that we get to formulate them and discuss them and when they figure out what would work best and what’s reasonable and what’s doable is really wonderful.*”  (P 01B)   - Poll reached mostly direct care providers   “*I like the poll was more direct clinicians than administrative managers. It looked like most of the respondents were clinicians and did not have administrative time.*” (M 07/22) |
| ***Drivers of***  ***Change and Stability*** | |  |  |
| **Sources of authority:**  Degree of (perceived) influence from political authority; economic authority; norms, beliefs, and values | | - Operation partners/memo driving change, not workgroup   “*I would say central office has 80 percent say.*”   - Operations partners would make the ultimate decision on guidelines   “*I think that you know if the guidelines were not in the direction the office wanted to go I think the office would have made the ultimate decisions of what had happened*.” (P 04F) | - Workgroup members believed they drafted clear, specific guidance   “*We want to have really clear this is what needs to be done by what measures need to be collected at what frequency and why is it important – why are we doing this? It helps as provide information to patient and guide sessions. We want to make sure this is clear guidance.*” (M 05/06)   - Operations partners expressed flexibility and concern for well-being of workgroup   “*We are more flexible and your mental health and getting a good product is important as well. Don’t challenge yourselves unnecessarily.*” (M 06/24)  • Collaborative partnership between workgroup and operation partners  “*I think the bulk of it is being informed by frontline providers but then ultimately the approval will come from central office which is also subject matter experts in the area of PTSD. So it’s not the recommendations are being driven by the field um but-so I would say both. 50-50, ultimately 50-50.*” (P 04B)   - Positive response from the field to the workgroup   “*I was on my PTSD mentoring call for [the region] earlier and I think they forgot that I am in the workgroup. They started just going on about the workgroup. They said isn’t it amazing that the MBC workgroup is doing all of this reach out? They are doing all of this polling and focus groups and really trying to get our opinion, and they gave all of this praise. They said, “Wouldn’t it be amazing if everything at the VA happened like this?*” (M 07/22) |
| **Social skills:**  Degree of use of tactics such as interpreting, framing, brokering, and bridging | |  | - Group Cohesion - All group members involved and frequently attended - All group members assisted with writing of guidelines - When unexpected changes to workgroup, different group members stepped in and took over tasks - Purposeful, minimal input from operations partner   “*In terms of priority, making sure you give us absolutely what we need, but can also give us what you think we need a relatively small amount of stuff we can send as guidance [...] You have a different perspective and that’s valued and it’s up to you where you go*.” (M 05/20)   - Group Consensus for poll - Group Consensus for focus group questions |
| **Exogenous shocks:**  Degree of stability or instability; changes in funding, legislation, or field actors | | - Challenges in MBC due to COVID-19 pandemic   “*[PTSD Clinical Team Director] was sharing with us her whole staff is working from home and how hard it’s been to collect measures.*” (M 05/27)   - Some workgroup members had lack of protected time for the workgroup   *“I think this was you know putting in some work outside of normal business hours or pulling from other places you know because of everything that was happening personally I found myself sometimes trying to play catch up or feeling like I wasn’t able to dedicate all the time that I wanted to*.*”* (P 02F) | - During COVID-19, workgroup collaboratively decided to continue with sensitivity towards the state of the field   “*I feel okay to move forward but be personable and sensitive when considering how to approach the field knowing that people are in a lot of different states.*” (M 05/06)   - Some workgroup members received protected time for workgroup   “*I have 20% of my time in the fiscal year was being bought out [by the PTSD mentoring program overall] and now 25% now so I think that helps when the work is your time with this project.*” *(P 05)*  “*[Supervisor] was totally in support of doing it basically allowed me to pull myself out of administrative and clinical work for those three days to get my writing portion of it done.*” (P 01F) |

*Note.* P = Participant from Baseline Interview; M = Meeting followed by date MM/DD; B = Baseline Interview; F = Follow up Interview.
